# Testosterone Deficiency Promotes Hypercholesteremia and Attenuates Cholesterol Liver Uptake via AR/PCSK9/LDLR Pathways

**DOI:** 10.1155/2022/7989751

**Published:** 2022-05-13

**Authors:** Yu Yuefeng, Lin Zhiqi, Chen Yi, Zhu Keyu, Wan Heng, Wang Yuying, Wang Ningjian, Yu Yuetian, Gu Xinjie, Zhang Yihao, Lu Yingli, Xia Fangzhen

**Affiliations:** Institute and Department of Endocrinology and Metabolism, Shanghai Ninth People's Hospital, Shanghai JiaoTong University School of Medicine, Shanghai 200011, China

## Abstract

**Background:**

Testosterone deficiency is reportedly correlated with an elevation of cholesterol in plasma, but the mechanism remains unclear. Our objective was to investigate the effects of testosterone deficiency on cholesterol metabolism and the corresponding molecular changes in vivo and in vitro.

**Methods:**

SD rats were randomized into three groups: sham-operated (SHAM), subtotal orchiectomized (SO), and orchiectomized (ORX) and fed for 8 weeks. HepG2 cells were cultured with medium containing testosterone with the final concentrations of 0, 10, 30, and 300 nM. Method of isotope tracing and fluorescence labelling was adopted to investigate cholesterol metabolism. Several key molecules of cholesterol metabolism were also analyzed.

**Results:**

SO and ORX rats displayed dysfunctional liver uptake of cholesterol. HepG2 cells incubated with testosterone of lower and excessive level exhibited reduced capacity of cholesterol uptake. Further investigation revealed that lack of testosterone induced increased proprotein convertase subtilisin/kexin type 9 (PCSK9) and decreased low-density lipoprotein receptor (LDLR) both in vivo and in vitro. Moreover, the androgen receptor (AR) antagonist flutamide mimicked the effects of testosterone deficiency on PCSK9 and LDLR indicating the role of AR as a mediator in triggering attenuating liver cholesterol uptake in which testosterone instead of dihydrotestosterone (DHT) is the major functional form of androgen.

**Conclusion:**

Testosterone deficiency attenuated cholesterol liver uptake mediated by the PCSK9-LDLR pathway, in which AR and testosterone without transforming to DHT play important roles.

## 1. Introduction

Risk of cardiovascular diseases (CVD) in men is about three times higher than that in women [1], explanations for which are varied. Persson et al. highlighted the athero-protective role of estrogen which makes females less vulnerable [2]. Similarly, testosterone has been considered to be associated with cholesterol metabolism. Some studies suggest that the physiologic testosterone level is adequate for increasing the risk of CVD related to cholesterol [3]. Contradictorily, there are research favoring testosterone deficiency as a factor of generating hypercholesteremia [4, 5]. In fact, aging men with late-onset hypogonadism (LOH) which is characterized by a decline of testosterone levels display increased levels of low-density lipoprotein (LDL) [6]. Nevertheless, how testosterone deficiency specifically affects the cholesterol metabolism still remains enigmatic.

Cholesterol homeostasis is maintained by the balance among several metabolic pathways including the de novo synthesis, intestinal absorption of cholesterol, and the liver uptake. The body gains cholesterol endogenously from the de novo synthesis rate, limited by 3-hydroxy-3-methylglutaryl coenzyme A reductase (HMGCR). Cholesterol is also gained exogenously through diet which is absorbed in the intestine where cholesterol is transcellularly transported by Niemann–Pick C1-like 1 (NPC1L1) and secreted back to the lumen by ATP-binding cassette subfamily G 5/8 (ABCG5/8) dimer. In contrast, cholesterol in plasma is removed from circulation mainly by the liver uptake which is essentially mediated by LDLR-induced endocytosis. PCSK9 escorts LDLR to lysosome for ubiquitination-related degradation [7].

Statins which have been proven efficacious by data of high quality for many years and recommended as the first-line therapy for CVD risk attenuating de novo synthesis of cholesterol. Subsequently, PCSK9 inhibitors and ezetimibe emerged against the backdrop of increasingly reported adverse effects of statins and the plight of refractory hypercholesteremia as alternative medications aimed at facilitating cholesterol liver uptake and reducing intestinal absorption, respectively [8]. Each of these drugs has distinctive functioning targets. However, previous studies of hypercholesteremia with testosterone deficiency remain superficial as most of them failed to identify the specific pathway of cholesterol metabolism in pathogenesis neither in vivo nor in vitro. This lack of knowledge is remarkably intriguing because by filling this gap, physicians are facilitated to tailor therapy plans for such a group of patients.

The goal of this research is to identify the specific pathway of cholesterol metabolism affected by testosterone deficiency and explore the molecular mechanisms involved. To achieve this, we adopted the methods of isotope tracing and fluorescence and identified attenuated cholesterol liver uptake as a pathogenic factor of hypercholesteremia in the state of testosterone deficiency both in animals and the cell line. We further revealed that PCSK9 upregulation and concomitant LDLR reduction are responsible for the impaired liver clearance of LDL in which testosterone acts as the functional form mediated by AR. These results provide new insights into the molecular basis of hypercholesteremia in testosterone deficient patients upon which precise treatment could be accomplished.

## 2. Materials and Methods

### 2.1. Animals

Male Sprague Dawley rats (8-week of age) were obtained from Shanghai Laboratory Animal Center in China. They were housed in pathogen-free facilities with a reversed 12 : 12-h light-dark cycle. The temperature was kept at 22 ± 2°C. Rats had access to a normal chow diet and water ad libitum. Body weights were measured weekly. All procedures were conducted in accordance with the guidelines of Animal Research Committee of Shanghai Ninth's People's Hospital affiliated with Shanghai Jiao Tong University School of Medicine and Institutional Animal Care and Use Committee (IACUC).

### 2.2. Orchiectomy

After one-week accommodation to the new environment, all rats were randomized into three groups: SHAM (*n* = 6), SO (*n* = 6), and ORX (*n* = 6). After anaesthetization and regular disinfection, orchiectomy was carried out as previously described [9]. In brief, skin around the scrotum was incised, and blood vessels nearby together with vas deferens were ligated followed by removal of the testes. The ORX group underwent bilateral testes removal, while one-fourth of the testis was left in the SO group. The sham operation was performed in which skin was incised and sutured. The sham group served as a baseline physiological control.

### 2.3. Cells Culture and Treatments

HepG2 cells were grown in low-glucose Dulbecco's modified Eagle's medium (DMEM) (Gibco, Waltham, US) supplemented with 12% charcoal-treated fetal bovine serum (FBS) (Biological Industries, Haemek, Israel), 100 U/mL penicillin, and 100 mg/mL streptomycin at 37°C in 5% CO_2_.

Testosterone (Adamas-Beta, Shanghai, China) dissolved in ethanol was added to the medium to achieve final concentrations of 0, 10, 30, and 300 nM [10] with a 0.1% (v/v) final volume of ethanol in the medium. Previous research [11, 12] had substantiated that this volume of ethanol did not intervene lipid composition. Cells were incubated with medium containing testosterone for 72 h. To antagonize the AR or inhibit 5*α* reductase, 1.5 *μ*M dutasteride or 50 *μ*M flutamide was dissolved in dimethyl sulfoxide (DMSO), respectively, with which cells were incubated for 48 h.

### 2.4. Biochemical Measurement and Metabolic Studies

Blood glucose was determined from tail vein blood using the Xpress glucometer (Nova Biomedical, Waltham, US). Serum quantification of total testosterone was measured in duplicate via a chemiluminescent microparticle immunology assay (CMIA) as previously described [9]. Serum quantification of TC, LDLc, and HDLc uses LabAssay Cholesterol kits (Wako, Osaka, Japan). Serum TG was determined with a Triglycerides Assay Kit (Jiancheng, Nanjing, China).

### 2.5. Body Composition Analysis

One week before sacrifice, all the rats were anesthetized by gas and scanned by CT using Quantum GX (Perkin Elmer). Images were analyzed, and three-dimensional models were reconstructed using Analyze 12.0 software.

### 2.6. Cholesterol Liver Uptake and Intestinal Absorption in Rats

Short-term acute cholesterol assay in vivo was applied as previously described [13]. At 20 weeks of age, each rat was treated with 200 *μ*L of corn oil added with 5 *μ*Ci ^3^H-cholesterol (Perkin Elmer, US) and 0.1 mg unlabeled cholesterol by gavage. 2 hours later, rats were euthanized. Plasma and tissues were taken. Radioactivity in samples was analyzed by liquid scintillation counting (Tri-Cab 4810TR, Perkin Elmer, CA, US) as previously described [14]. The percentage cholesterol intestinal absorption was calculated as follows: % cholesterol absorption = (^3^H in plasma/^3^H dosing) × 100. Also, the percentage cholesterol liver uptake was calculated as follows: % cholesterol uptake = (3H in liver / 3H dosing) × 100. To calculate the total ^3^H in plasma, we assumed that each rat possesses 4.0 ml plasma per 100 g body weight.

Cholesterol absorption was also measured by the plasma dual isotope ratio method as previously described [13]. In brief, rats were fast for 12 hours before the injection of 5 *μ*Ci ^3^H-cholesterol in 0.9% NaCl via tail veins and gavage with 5 *μ*Ci ^14^C-cholesterol dissolved in skim milk. After a 6-hour fast, the rats were accessible to food and water ad libitum. 72 hours after the administration of radiolabeled cholesterol, rats were sacrificed. Plasma was harvested. Radioactivity was analyzed by liquid scintillation counting (Tri-Cab 4810TR, Perkin Elmer, CA, US) as previously described [14]. The percentage cholesterol absorption was calculated as follows: % cholesterol absorption = (percentage of intragastric dose [^14^C] cholesterol per ml plasma/percentage of intravenous dose ^3^H cholesterol per ml plasma)^*∗*^100.

### 2.7. Cholesterol Biosynthesis Assay

Cholesterol biosynthesis experiment was applied as previously described [15, 16]. Cells were treated with medium supplemented with 0.3 *μ*Ci/ml ^14^C-acetate sodium (Perkin Elmer, CA, US). 24 hours later, cells were harvested, and the total lipids were extracted using 10%KOH/95% ethanol (v:v 1 : 10). Then, total cholesterol was isolated from total lipids using a solvent mixture of petroleum ether/diethyl ether/acetate acid (v:v:v 80 : 30 : 1). Radioactivity was determined by liquid scintillation counting (Tri-Cab 4810TR, Perkin Elmer, CA, US). The results are presented as follows: [^14^C] in extracted cholesterol (*μ*Ci)/dry cell weight (mg).

### 2.8. Cholesterol Liver Uptake in Cells

Cholesterol liver uptake in cell was carried out according to the manufacturer's guide HepG2 cells were incubated with 15 *μ*g/mL of BODIPY-labeled LDL (Invitrogen, Waltham, US) for 2 h at 37°C. Then, cell slides were then incubated with DAPI (Sigma-Aldrich, Missouri, US) for 5 min. Images were acquired using an Olympus microscope. Fluorescence density was analyzed using ImageJ software.

### 2.9. Western Blot

Cell and liver protein samples were extracted and prepared as previously described [17]. Proteins were probed using the anti-HMGCR (1 : 1000; Novus), anti-ABCG5 (1 : 500; Proteintech), anti-NPC1L1 (1 : 1000, Novus), anti-PCSK9 (1 : 1000; Proteintech), and anti-LDLR (1 : 1000; Proteintech) and revealed using the horseradish peroxidase (HRP) conjugated secondary antibodies (1 : 2000, Cell Signaling Technology) and HRP-conjugated beta actin antibody (1 : 2000, Proteintech). The housekeeping protein beta-actin was used as an internal reference. Blots were visualized by enhanced chemiluminescence. Densitometry quantification of signals was conducted by ImageJ software.

### 2.10. Quantitative Real-Time PCR

Total RNA was extracted from tissue samples using TRIzol reagent. RNA concentrations were measured using Nanodrop (Allsheng, Hangzhou, China). cDNA was synthesized using a PrimeScript TMRT Master Mix (Takara, Shiga, Japan) according to manufacturer's instructions. qPCR was performed with LightCycler 96 (Roche, Basel, Swiss) using the Hieff qPCR SYBR Green Master Mix (Yeasen, Shanghai, China) according to the guidelines of the kits. The detailed qRT-PCR protocol has been described previously. Beta-actin served as the housekeeping gene. The primer sequences used are listed in Table 1.

## 3. Results

### 3.1. Testosterone Deficiency Leads to Hypercholesteremia

**Figure 1:**
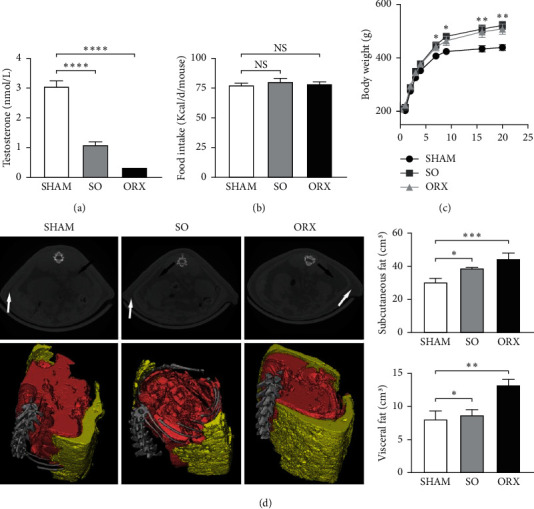
Metabolic characteristics of sham-operated (SHAM), subtotal orchiectomized (SO) and orchiectomized (ORX) rats. (a) Serum testosterone level six weeks after operations. Rats in SO and ORX groups displayed markedly reduced testosterone levels as expected. Testosterone level of rats in ORX group were all under the lower limit of the measurement (b) Food intake of the rats during the experimentation. (c) The body weight alteration of rats within 20 weeks. (d) Representative graphs of computed tomography (CT) scanning and three-dimensional reconstructed models (yellow: subcutaneous fat; red: visceral fat) of SHAM, SO and ORX group. Volume of subcutaneous and visceral fat are analyzed from the images. Black and white arrows indicates visceral and subcutaneous fat of SHAM, SO and ORX rats respectively. Data are presented as mean ± SEM. Statistical analyses are unpaired *t*-test or one-way ANOVA. ^*∗*^*P* < 0.05; ^*∗∗*^*P* < 0.01; ^*∗∗∗*^*P* < 0.001; ^*∗∗∗∗*^*P* < 0.0001 ns: non-significant.

It has been substantiated that patients with hypercholesteremia suffer from higher cardiovascular risk. To examine the effects of testosterone deficiency on cholesterol homeostasis and cardiovascular diseases, we used testosterone-deficient SD rats having undergone orchiectomy and subtotal orchiectomy, respectively. As expected, testosterone level six weeks after the operation in the rats of both groups markedly reduced compared with the sham-operated (SHAM) group (Figure 1(a)).

**Figure 2:**
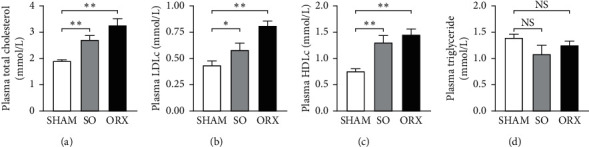
Testosterone deficiency induced disorder of cholesterol metabolism. (a) Plasma total cholesterol (TC), (b) low-density lipoprotein cholesterol (LDLc), (c) high density lipoprotein cholesterol (HDLc), (d) triglyceride (TG) of the rats. Data are presented as mean ± SEM. Statistical analyses are unpaired *t*-test or one-way ANOVA. ^*∗*^*P* < 0.05; ^*∗∗*^*P* < 0.01; ^*∗∗∗*^*P* < 0.001; ns: non-significant.

Although the food intake was similar among three groups (Figure 1(b)), testosterone-deficient rat displayed increased body weight (Figure 1(c)). Besides, computed tomography (CT) scanning and three-dimensional reconstruction revealed that SO and ORX rats gained more subcutaneous and visceral fat than the SHAM groups (Figure 1(d)). These results showed that rats with testosterone deficiency experienced metabolic disorder. Moreover, total cholesterol (TC), low-density lipoprotein cholesterol (LDLc), and high-density lipoprotein cholesterol (HDLc) levels in the SO and ORX group were higher than those observed in the SHAM group (Figures 2(a)–2(c)). However, no significant difference of triglyceride (TG) was observed among the three groups (Figure 2(d)). Taken together, these data suggest that testosterone deficiency triggers hypercholesteremia in rats.

### 3.2. Testosterone Deficiency Attenuates the Liver Uptake of Cholesterol

Cholesterol homeostasis depends on exogenous absorption in intestine, uptake and de novo synthesis of the liver, and abnormality in either of which can lead to dyslipidemia in clinical cases.

**Figure 3:**
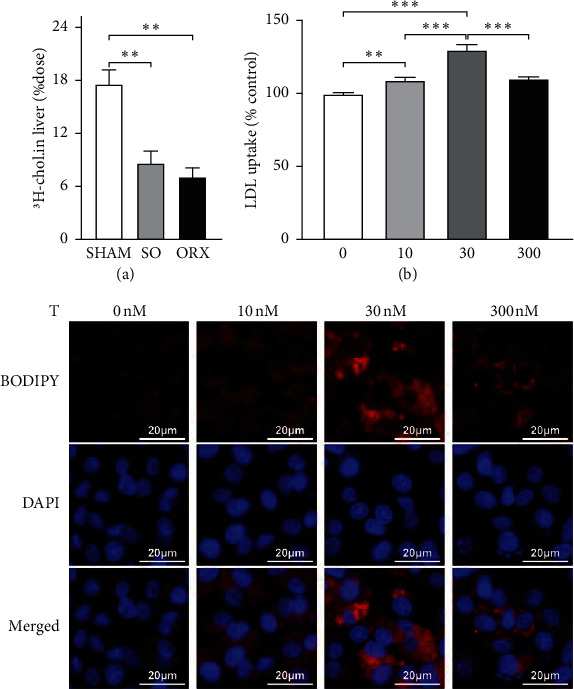
Testosterone deficiency attenuates cholesterol liver uptake. (a) Amount of 3H-cholesterol in the liver of rats 2 hours after the gavage with 5 μCi 3H-cholesterol and 0.1 mg unlabeled cholesterol demonstrating the liver cholesterol uptake (*n* = 6). (b) Cholesterol uptake of HepG2 cells in different levels of testosterone. Cells were incubated with 0, 10, 30, 300 nM testosterone for 72 h and then BODIPY-labeled low-density lipoprotein (LDL) (red) for 2 h. After wash of PBS for twice, cells were stained with DAPI (blue) and visualized using confocal microscopy. Data are presented as mean ± SEM. Statistical analyses are unpaired *t*-test or one-way ANOVA. ^*∗*^*P* < 0.05; ^*∗∗*^*P* < 0.01; ^*∗∗∗*^*P* < 0.001; ns: non-significant.

By orally administering 5 *μ*Ci ^3^H-cholesterol, we observed no difference of cholesterol intestinal absorption among SHAM, SO, and ORX rats (Figure S1a). The plasma dual-isotope ratio method also demonstrated a similar result (Figure S1b). However, 2 h after the treatment of 5 *μ*Ci ^3^H-cholesterol and 0.1mg unlabeled cholesterol by gavage, SO and ORX rats showed lower liver uptake of cholesterol (Figure 3(a)). Moreover, we incubate HepG2 with 0, 10, 30, and 300 nM testosterone and conducted the liver uptake assay. Fluorescence analysis revealed an increase in labelled LDL uptake upon testosterone treatment of higher concentration (30 nM) compared with lower testosterone levels (10 nM) and control (0 nM). Intriguingly, when further increasing testosterone to 300 nM, the cholesterol uptake in HepG2 was impeded (Figure 3(b)). Subsequent investigation of de novo synthesis of HepG2 using ^14^C-sodium acetate demonstrated that rate of cholesterol synthesis was similar in different testosterone levels (Figure S1c). These data indicate that instead of intestinal absorption and the de novo synthesis, testosterone deficiency triggers hyperlipidemia by hampering physiological liver uptake of cholesterol.

### 3.3. Testosterone Deficiency Reduces the Liver Uptake of Cholesterol via the PCSK9-LDLR Pathway

To further elucidate potential mechanism of cholesterol metabolism disorder under low testosterone levels, we performed western blotting and quantitative real-time PCR (qPCR) on various key regulators in cholesterol homeostasis.

**Figure 4:**
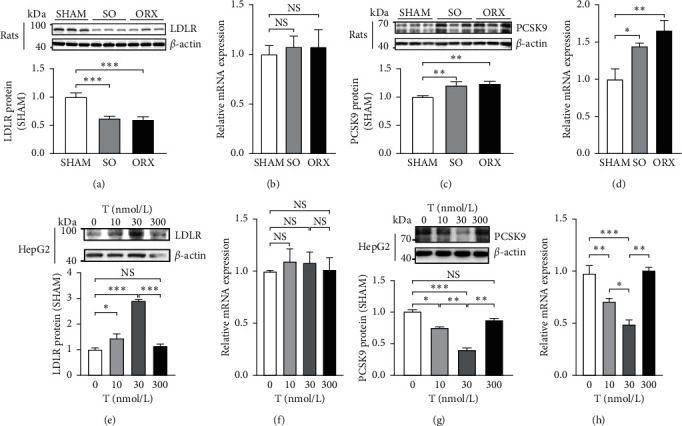
Testosterone deficiency reduces low-density lipoprotein receptor (LDLR) in liver by upregulating proprotein convertase subtilisin/kexin type 9 (PCSK9). Key mediators of liver cholesterol uptake LDLR and PCSK9 were analyzed in vivo and in vitro in different conditions of testosterone by western blotting and real-time quantitative PCR. ((a), (b)) LDLR and ((c), (d)) PCSK9 level in the liver of SHAM, SO, ORX rats. ((e), (f)) LDLR and ((g), (h)) PCSK9 level in HepG2 cells incubated with 0 (control), 10, 30, 300 nM testosterone for 72 h. Data are presented as mean ± SEM. Statistical analyses are one-way ANOVA. ^*∗*^*P* < 0.05; ^*∗∗*^*P* < 0.01; ^*∗∗∗*^*P* < 0.001; ns: non-significant.

To investigate the molecular changes resulting in liver uptake inadequacy in the testosterone-deficient state, key mediators involved in the liver uptake were studied. Both SO and ORX rats demonstrated lower LDLR protein content in liver than that in the SHAM rats (Figure 4(a)). Intriguingly, the mRNA levels of LDLR were similar among three groups (Figure 4(b)) indicating that posttranslational regulation might have involved. Therefore, we conducted western blotting and qPCR to PCSK9, a crucial posttranslational regulator of LDLR. It is revealed that both liver PCSK9 mRNA and protein levels were elevated in the SO and ORX group compared with the SHAM group (Figures 4(c) and 4(d)). We next examined these alterations in vitro, and similar results were observed. HepG2 were cultured in medium with different testosterone levels. When testosterone rose from 0 nM, 10 nM to 30 nM, LDLR was upregulated with no significant change in mRNA levels, while PCSK9 was downregulated. Strikingly, consistent with the previous liver uptake assay in 300 nM testosterone, PCSK9 increased and LDLR content dropped accordingly (Figures 4(e)–4(h)). Together, these data demonstrate that testosterone deficiency increases the PCSK9 expression and thus exacerbates the degradation of LDLR, leading to inadequate cholesterol liver uptake and further higher risk of hypercholesteremia. Furthermore, excessive testosterone produces deleterious effect on cholesterol liver uptake.

### 3.4. Androgen Receptor (AR) Is a Key Regulator and Testosterone is the Functional Form in Testosterone-Induced Cholesterol Liver Uptake Changes

**Figure 5:**
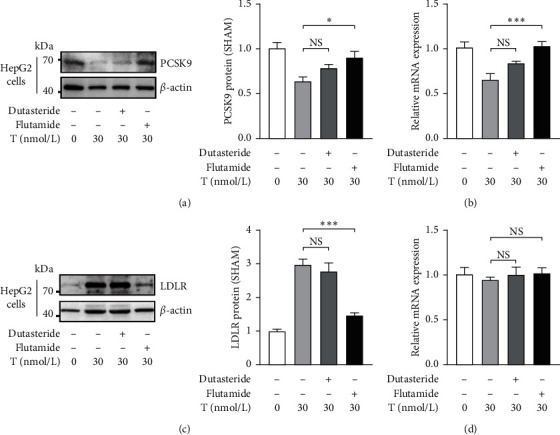
Androgen receptor (AR) antagonist flutamide abolishes the testosterone-induced PCSK9 downregulation and LDLR upregulation while 5*α* reductase inhibitor dutasteride shows no effect. HepG2 cells were incubated with 0, 30 nM testosterone for 72 h or 30 nM testosterone plus 50 *μ*M AR antagonist flutamide or 1.5 *μ*M 5*α* reductase inhibitor dutasteride for 72 h. Then cells were harvested. ((a), (b)) PCSK9 and ((c), (d)) LDLR were analyzed by western blotting or real-time quantitative PCR. Data are presented as mean ± SEM. Statistical analyses are one-way ANOVA. ^*∗*^*P* < 0.05; ^*∗∗*^*P* < 0.001; ^*∗∗∗*^*P* < 0.01; ns: non-significant.

Physiological effects of testosterone are mainly mediated by AR. To examine the role of AR in the testosterone-dependent altered cholesterol liver uptake, HepG2 was cultured with 30 nM testosterone plus 50 *μ*M AR antagonist flutamide and displayed PCSK9 elevation (Figures 5(a) and 5(b)) and concomitant LDLR reduction similar to the control (Figure 5(c)) indicating AR as a mediator of testosterone effect on the liver uptake. Besides, mRNA levels of LDLR were similar among different groups (Figure 5(d)). To further investigate either testosterone or the more active dihydrotestosterone (DHT) involve in this process, we incubated HepG2 with 30 nM testosterone plus 1.5 *μ*M 5-*α* reductase inhibitor dutasteride and observed no alteration of PCSK9 (Figures 5(a) and 5(b)) and LDLR levels (Figures 5(c) and 5(d)) compared with cells without dutasteride supplementation. Together, these data reveal that AR's activation by testosterone may be responsible for androgen-induced alteration of cholesterol liver uptake.

## 4. Discussion

In this study, we have shown that testosterone deficiency impaired liver uptake of cholesterol which results in increased total cholesterol and LDLc in circulation, potential mechanism of which is lower LDLR in hepatocytes associated with upregulated PCSK9. This process is induced by AR activation in which testosterone acts as a functional form. Nevertheless, excessive testosterone supplementation contrarily decreases the liver uptake.

We found that total cholesterol and LDLc level in SO and ORX rats were significantly higher than that in the SHAM group. Moreover, abdominal aortae staining also indicates higher cardiovascular risk in testosterone-deficient rats. These data are in agreement with a number of clinical studies. In fact, low testosterone is closely associated with increased levels of LDLc [18]. Besides, testosterone supplementation is reported to decrease circulating LDLc [19]. Significantly increased HDLc level was also observed in testosterone-deficient rats, which is consistent with the previous study [20]. But changes in serum HDLc by testosterone deficiency may not reflect changes in HDL function [20].

The body gains cholesterol either endogenously from de novo synthesis or exogenously from intestinal absorption and mainly clears cholesterol by liver uptake [21]. Our study suggests that low testosterone impedes the liver uptake, while it shows no effect on de novo synthesis and intestinal absorption in rats. Further testosterone supplementation to 30 nmol/L (physiological concentration of testosterone) facilitated hepatocytes to take up more cholesterol in vitro. Specifically, in the process of liver uptake, LDLR and PCSK9 are two crucial regulators. In the present study, we have demonstrated that testosterone deficiency decreased LDLR in hepatocytes, while its mRNA level remains unchanged. Moreover, PCSK9 increased in testosterone-deficient condition. This indicates that low testosterone may trigger excess of PCSK9 which posttranslationally decreases LDLR. Consequently, abnormal clearance of LDLR leads to inadequate LDL removal in plasma. Mechanism beneath PCSK9's activation may include the classical SREBP2 pathway which is potentiated by hepatocyte nuclear factor 1*α* (HNF1*α*) and mediated by histone 1 nuclear factor P (H1NFP) [22] or newly reported gene rearrangement associated with hepatocyte nuclear factor 4*α* (HNF4*α*) [23]. Further studies are needed to elucidate the confined regulation of PCSK9 involved.

Excessive testosterone has deleterious effects on the cardiovascular system. Our data showed that excessively high testosterone attenuates cholesterol uptake of hepatocytes in which PCSK9-LDLR alteration similar to testosterone deficiency was observed. It is presumably associated with accompanied hyperhomocysteinemia in patients with hyperandrogenemia [24]. In fact, the abuse of high-dose synthetic steroids is an independent risk factor for coronary heart disease [25]. Against the backdrop of exponentially increased prescriptions of testosterone for age-related hypogonadism [26], fully assessing the risk and optimal dosage of androgen regimen before the replacement therapy would mitigate the side effect and benefit the patients for long run.

Testosterone is transformed into DHT which is catalyzed by 5*α* reductase. Testosterone and DHT are both ligands to activate AR followed by the cascade in the downstream. In this study, we found that AR antagonist flutamide can abolish the effect of testosterone while 5-*α* reductase cannot, which indicates the crucial role of AR in testosterone-induced modulation of liver cholesterol uptake. Testosterone activates AR either located on the membrane which triggers nongenomic, rapid cascade, or on the nuclear which mediates the genomic effect by regulating transcription factors [27]. Besides, novel extranuclear AR action on metabolism homeostasis is also reported [28]. Elaborated links between AR activation and PCSK9 regulation deserve in-depth studies. No one has reported that testosterone can directly affect PCSK9. And, the existing literature reported that there was no direct binding between AR and PCSK9. However, by exploring the downstream of androgen-AR pathway and the upstream of PCSK9-LDLR pathway, we found that there may be potential protein interaction. Levy et al. found that AR regulates catenin beta-1 (CTNNB1) [29]. Furthermore, CTNNB1 interacts with epidermal growth factor (EGF) [30]. EGF has been reported to have the potential to inhibit PCSK9 [31]. Testosterone might interact indirectly with PCSK9 which might be driven by AR.

Statins have been strongly substantiated to ameliorate or prevent atherosclerotic cardiovascular diseases (ASCVD) by lowering LDLc levels and universally considered as the first choice of lipid lowering [32]. However, as the clinical emphasis of personalized therapy when statins are applied to hypogonadal individuals, increasing bodies of evidence have captured attention to their side effects. In fact, statins were reported to exacerbate androgen deficiency by reducing peripheral ARs [33] or directly lowering serum testosterone levels [34] due to Leydig cells' selective uptake of cholesterol from the de novo synthesis as ingredients of steroidogenesis [35], which raises the urgent need of an alternate therapy. Our study accentuates the role of PCSK9 in the pathogenesis of hypercholesteremia in testosterone-deficient individuals indicating potential value of PCSK9 inhibitors when targeting the hypogonadal patients. Indeed, PCSK9 inhibitors display no detrimental effect on the testosterone level compared with statins in the previous study [36]. Efficacy of PCSK9 inhibitors specifically men with declined testosterone level awaits confirmation in future studies.

In conclusion, we have provided the first evidence to the fact that attenuated cholesterol liver uptake caused by the PCSK9-LDLR pathway is responsible for hypercholesteremia in the state of testosterone deficiency, in which AR and testosterone without transformation to DHT are required. These findings have pharmacological and clinical implications for tailored therapy of hypercholesteremia in aging men.

## Figures and Tables

**Table 1:** Primer sequences for real-time quantitative RT-PCR.

Gene name	Species	Sequence		Size (bp)	GenBank accession no.
*β*-actin	Human	Forward	5′-GCCGCCAGCTCACCAT-3′	171	NM001101
		Reverse	5′-AGGAATCCTTCTGACCCATGC-3′		

*β*-actin	Rat	Forward	5′-GATATCGCTGCGCTCGTCG-3′	217	NM031144
		Reverse	5′-CAATGCCGTGTTCAATGGGG-3′		

LDLR	Human	Forward	5′-ATTGTCCTCCCCATCGTGCT-3′	164	NM000527
		Reverse	5′-CTGTAGCCGTCCTGGTTGTG-3′		

Ldlr	Rat	Forward	5′-TTGGCTGCATCAATGTGACC-3′	155	NM175762
		Reverse	5′-CAAGCACTCGTTGGTCTTGC-3′		

PCSK9	Human	Forward	5′-GGTTAGCGGCACCCTCATAG-3′	231	NM174936
		Reverse	5′-CCAACTGTGATGACCTCGGG-3′		

Pcsk9	Rat	Forward	5′-AAGATAGCTCCCCTGACGGA-3′	170	NM199253
		Reverse	5′-ATGGCTGTCACACTTGCTCG-3′		

LDLR/Ldlr, low-density lipoprotein receptor; PCSK9/Pcsk9, proprotein convertase subtilisin/kexin type 9.

## Data Availability

The data that support the findings of this study are available from the corresponding author upon reasonable request.

## Supplementary Materials

Supplementary Materials Figure S1: testosterone showed no effects on the intestinal absorption and de novo synthesis of cholesterol. (a) Amount of ^3^H-cholesterol in plasma of rats after gavage with 5 *μ*Ci ^3^H-cholesterol for 2 hours indicating the intestinal absorption of cholesterol (*n* = 6). (b) Cholesterol absorption in three groups of rats determined by plasma dual-isotope ratio method 72 hours after injecting ^3^H-cholesterol via tail veins and orally administering ^14^C-cholesterol (*n* = 6). (c) The rate of cholesterol biosynthesis in HepG2 cells cultured with testosterone of different concentrations. Cells were incubated with 0, 10, 30, and 300 nM testosterone for 72 h followed by supplementation of 0.3 *μ*Ci/ml ^14^C-acetate sodium. 24 hours later, cholesterol was extracted, and radioactivity was analyzed. Data are presented as mean ± SEM. Statistical analyses are unpaired *t*-test or one-way ANOVA. ^*∗*^*P* < 0.05; ^*∗∗*^*P* < 0.01; and ^*∗∗∗*^*P* < 0.001; ns: nonsignificant.
