# Supplementary material for: Testosterone Deficiency Promotes Hypercholesteremia and Attenuates Cholesterol Liver Uptake via AR/PCSK9/LDLR Pathways
Source: Int J Endocrinol. 2022 May 13;2022:7989751. doi: 10.1155/2022/7989751 (PMC9122719; doi:10.1155/2022/7989751)
Supplement: Supplementary Materials — Figure S1: testosterone showed no effects on the intestinal absorption and de novo synthesis of cholesterol. (a) Amount of 3H-cholesterol in plasma of rats after gavage with 5 μCi 3H-cholesterol for 2 hours indicating the intestinal absorption of cholesterol (n = 6). (b) Cholesterol absorption in three groups of rats determined by plasma dual-isotope ratio method 72 hours after injecting 3H-cholesterol via tail veins and orally administering 14C-cholesterol (n = 6). (c) The rate of cholesterol biosynthesis in HepG2 cells cultured with testosterone of different concentrations. Cells were incubated with 0, 10, 30, and 300 nM testosterone for 72 h followed by supplementation of 0.3 μCi/ml 14C-acetate sodium. 24 hours later, cholesterol was extracted, and radioactivity was analyzed. Data are presented as mean ± SEM. Statistical analyses are unpaired t-test or one-way ANOVA. ∗P < 0.05; ∗∗P < 0.01; and ∗∗∗P < 0.001; ns: nonsignificant. [file 7989751.f1.docx]

Figure S1


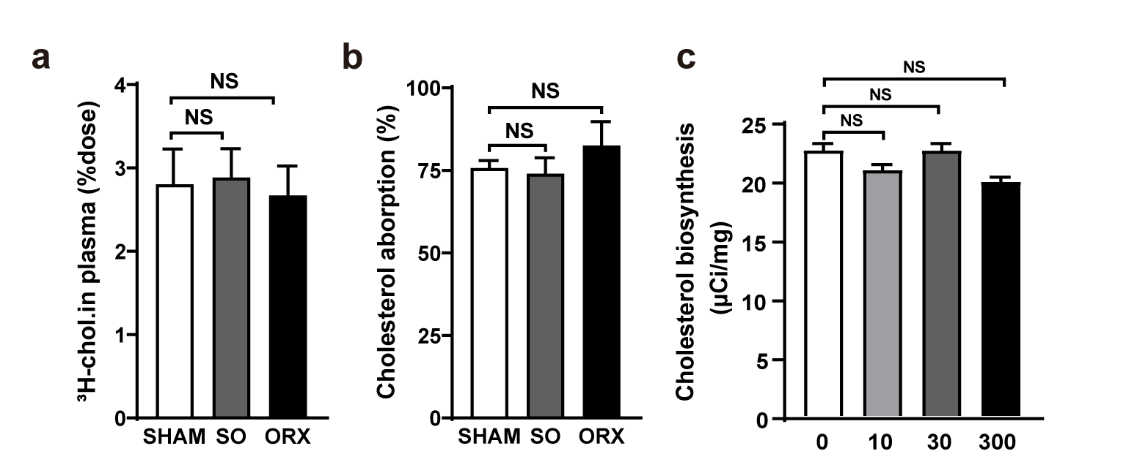


**Figure S1 Testosterone showed no effects on the intestinal absorption and de nevo synthesis of cholesterol**. **(a)** Amount of ^3^H-cholesterol in plasma of rats after gavage with 5 µCi ^3^H-cholesterol for 2 hours indicating the intestinal absorption of cholesterol (n=6). **(b)** Cholesterol absorption in three groups of rats determined by plasma dual-isotope ratio method 72 hours after injecting ^3^H-cholesterol via tail veins and orally administering ^14^C-cholesterol (n=6). **(c)** The rate of cholesterol biosynthesis in HepG2 cells cultured with testosterone of different concentrations. Cells were incubated with 0, 10, 30, 300 nM testosterone for 72 h followed by supplementation of 0.3 μCi /ml 14C-acetate sodium. 24 hours later cholesterol were extracted and radioactivity were analyzed. Data are presented as mean ± SEM. Statistical analyses are unpaired t-test or one-way ANOVA. *P<0.05; **P<0.01; ***P<0.001; ns: non-significant
